# Structure Learning in Human Sequential Decision-Making

**DOI:** 10.1371/journal.pcbi.1001003

**Published:** 2010-12-02

**Authors:** Daniel E. Acuña, Paul Schrater

**Affiliations:** 1 Department of Computer Science and Engineering, University of Minnesota, Minneapolis, Minnesota, United States of America; 2 Department of Psychology, University of Minnesota, Minneapolis, Minnesota, United States of America; John Radcliffe Hospital, United Kingdom

## Abstract

Studies of sequential decision-making in humans frequently find suboptimal performance relative to an ideal actor that has perfect knowledge of the model of how rewards and events are generated in the environment. Rather than being suboptimal, we argue that the learning problem humans face is more complex, in that it also involves learning the structure of reward generation in the environment. We formulate the problem of structure learning in sequential decision tasks using Bayesian reinforcement learning, and show that learning the generative model for rewards qualitatively changes the behavior of an optimal learning agent. To test whether people exhibit structure learning, we performed experiments involving a mixture of one-armed and two-armed bandit reward models, where structure learning produces many of the qualitative behaviors deemed suboptimal in previous studies. Our results demonstrate humans can perform structure learning in a near-optimal manner.

## Author Summary

Every decision-making experiment has a structure that specifies how rewards are obtained, which is usually explained to the subject at the beginning of the experiment. Participants frequently fail to act as if they understand the experimental structure, even in tasks as simple as determining which of two biased coins they should choose to maximize the number of trials that produce “heads”. We hypothesize that participants' behavior is not driven by top-down instructions—rather, participants must learn through experience how the rewards are generated. We formalize this hypothesis using a fully rational optimal Bayesian reinforcement learning approach that models optimal structure learning in sequential decision making. In an experimental test of structure learning in humans, we show that humans learn reward structure from experience in a near optimal manner. Our results demonstrate that behavior purported to show that humans are error-prone and suboptimal decision makers can result from an optimal learning approach. Our findings provide a compelling new family of rational hypotheses for behavior previously deemed irrational, including under- and over-exploration.

## Introduction

From a squirrel deciding where to bury its nuts to a scientist selecting the next experiment, all decision-making organisms must balance exploration of alternatives against exploitation of known options in developing action plans. Finding a balance is equivalent to knowing when you can profit from learning about new options and knowing when you know enough. However, determining when exploration is profitable is itself a decision problem that requires understanding or learning about the statistical structure of the environment. Theoretical work on optimal exploration [1], [2] shows that assessing the long-term value of exploration involves integrating the predicted informational value of exploration with primary reward. Predicting the value of future information requires having a model of the reward generation process for the domain.

The structure learning problem may be present in tasks with as few as two options. Suppose, for example, that you interact with the environment by choosing one of the two options at discrete choice points and that the option chosen generates a stochastic binary reward. As a rational agent, your aim is to maximize the total reward from the environment, but the difficulty is that the rate of reward for each option is unknown and must be learned. In this simple setting, there may be several hypothesis about how the reward generation process works—how actions, observations and unknowns are *structurally* “connected.” We propose three kinds of structures that capture several versions of sequential decision-making tasks available in the literature. The first structure has temporal dependency between the present probability of reward and the past probability of reward, investigated in the context of *Multi-Armed Bandit problems* [3]–[5]. When this dependency involves a random walk, the environment becomes non-stationary and a rational agent will discount both past reward observations [6] and potential future reward (equivalent to discounting) and it will exhibit a higher learning rate in the sense of a greater dependence on recent reward information. In the second structure, reward probabilities can be affected by actions. For example, choosing an option may temporarily decrease the reward probability. Different kinds of action-reward probability contingencies can produce a range of different rational responses, from probability matching (foraging) to maximization. The third structure is *reward coupling* and is the primary focus of this paper.

**Figure 1:**
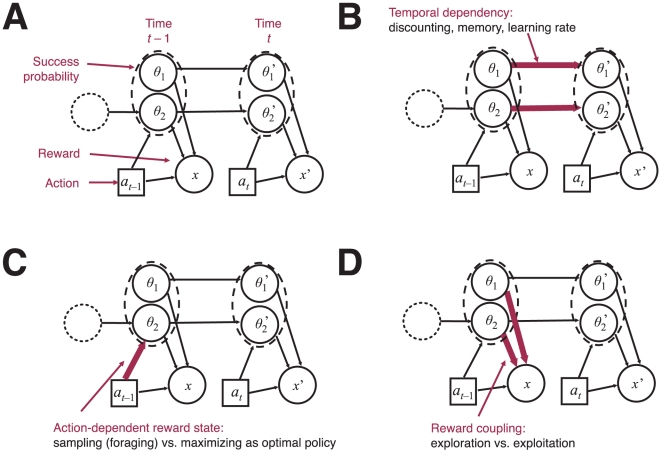
Different structures in sequential decision-making. **A**) General structure. Arcs highlighted denote **B**) temporal dependency between success probabilities, **C**) action-dependent reward state leading to different optimality principles—from foraging to maximization and **D**) reward coupling affecting exploration vs. exploitation demands.

To illustrate what structure learning entails, Fig. 1A shows a probabilistic graphical model representing the possible relationships between variables for a typical sequential decision task with two outcomes. In the graph, nodes represent unknown or observable quantities and links represent statistical contingencies between them. The unknown probabilities of reward at a given time 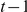 for both option 1 and 2 are represented by 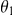 and 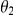, respectively. Taking action 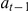 at time 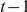 produces a reward 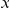 that can be either 0 (failure) or 1 (success). Learning the success probabilities must be balanced with the desire to maximize expected future reward. Different assumptions about the connectivity (structure) between variables produce a surprising range of rational responses. One of those structures is *temporal dependency* (see Fig. 1B) between success probabilities. In this case, rather than being fixed, the success probabilities 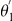 and 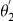 depend on past values 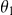 and 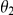 [3], [4]. The second structure includes an effect of actions on reward probabilities (see Fig. 1C). Different kinds of action-reward probability contingencies can produce a range of different rational responses, from matching to maximization [7], [8]. Fig. 1D illustrates *Reward coupling* which determines whether the reward probabilities are related to each other. For example, options may be probabilistically coupled so that if one option is “good” the other must be “bad”. This type of structure has profound consequences on exploratory and exploitative behavior.

To illustrate *reward coupling*, imagine you are serving a ball in tennis against an opponent who almost always adopts the same position near the center of the court. How do you choose whether you serve left or right? Assume the defender must anticipate and make its choice to defend left or right before it sees your serve. Clearly you should take advantage of the previous history of successful and unsuccessful serves against this opponent to try to exploit any weakness, but how you should make use of this history depends on what you can learn from your choices. For example, if you last served left and failed, can you infer it would have been better to serve right? The answer depends critically on the way options are probabilistically related. The outcomes of an anticipatory defender are probabilistically coupled - its probability of selecting left is one minus its probability of selecting right (similar to a coin flip). For coupled outcomes, what can be learned on each trial is independent of your actions and no active exploration is needed.

Imagine instead you throw a ball at one of two targets: left or right—with the goal of determining which target is easier to hit. In this case, you can infer little from a failure on the left target about your success on the right. The options are independent, which means that observing one option tells you little or nothing about the other. Exploration is then necessary for learning, and your choices impact what can be learned. Thus, the kind of probabilistic dependence between options determines whether passive (action independent) or active learning strategies are needed.

An organism with initial ignorance about the environment will not have a model of the probabilistic coupling, and thus will not know the value of exploration. But how can it know what kind of probabilistic dependence is present?

In this work, we investigate the possibility that people learn models of reward generation using rational analysis. From a rational perspective, actions should be selected both to increase reward and to provide information about the reward generation process. Probabilistic methods for learning dependencies between variables are termed structure learning or causal learning, and has been an active topic within the machine learning community. We argue that structure learning plays a major role in human sequential decision making. Because structure denotes the statistical relationships between entities and events, it forms the basis for generating future predictions, and it enables model-based approaches to reinforcement learning.

Using model-based (Bayesian) reinforcement learning [9]–[12] optimal exploration can be extended to handle uncertainty across a set of plausible reward generation models. In one formulation we follow here, latent parameters on model structure are treated as a hidden state, such that the algorithm tries to find values of the hidden state that maximize expected discounted reward. In essence, at the beginning of a set of tasks, we assume there is initial uncertainty over a parametric family of structures—causal models of reward generation. The learning of this causal structure is then incorporated into acting. This is a natural extension of causal induction (predictive of behavior in simpler tasks [13]) to sequential experimentation.

To maximize the differences that uncertainty about the causal relationships between options would produce, we exposed subjects to one of two possible models that represent two extremes in the exploration– exploitation trade-off in a slot-machine gambling environment, where the probabilistic coupling between the payoffs between machines must be learned. Using Bayesian RL to generate an optimal exploratory agent for this environment, we show that optimal actions with reward model uncertainty include exploratory actions that are specific to model learning, and exhibit patterns that would be considered over- and under- exploration for an agent without reward model uncertainty. We demonstrate that humans are able to learn the probabilistic coupling structure for this environment, and that they exhibit exploratory choice behavior predicted by reward model learning.

## Results

Participants made decisions in a set of 32 two-option tasks, each terminating stochastically, with an average of 48 trials. For each task, an option produced an stochastic binary reward with a fixed probability that had to be estimated by the participant. Participants were asked to maximized their reward gathered for the whole experiment and were compensated in proportion to the total reward.

Formally, the choice of option 1 or 2 transitions the agent into that state, and generates an observable binary reward 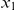 and 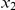, respectively. The reward distributions are initially unknown but remain constant within a task, which ends stochastically with a probability 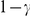. At the end of each task the reward distributions are reset. The tasks are analogous to playing slot machines in a casino. There are two slot machines. The state of the environment 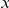 represents which of the slot machines is active. Actions involve selecting which of the machines to activate (pull the slot machine lever), and active machines generate binary rewards probabilistically.

To experimentally test how well humans can learn the probabilistic coupling structure of an environment, we used two environments with different reward structure designed to generate clear differences in decisions and exploratory behavior. In the first environment, which we term *independent*, the reward distributions for each machine are independent. In the second environment, called *coupled*, the two reward distributions are coupled by sharing a common cause: when one option gives reward, the other will not. The optimal policies for these environments generate exploratory behavior that span the range of possibilities, from independent where exploration is necessary to coupled, where exploration is superfluous. An agent with uncertainty about whether the environment is coupled or independent will need to learn both the coupling structure and the reward values of the options.

The environments were presented as two distinctive “blocks” of tasks. Each block was presented as a “game room” and machines in that game room had a unique color (blue in one room and yellow in the other). Unknown to the subjects, however, the first block of 16 tasks corresponded to one reward structure and the second block of 16 tasks corresponded to other reward structure.

We argue that it would be unreasonable for participants to assume a reward structure beforehand. They, instead, have to perform an estimation of this structure through a block of tasks while jointly learning the reward rates within the task. To predict human decisions in the task, we develop a normative model that makes decisions while actively gathering evidence about both task structure and the rewards available at each option and compare its performance both to other normative models that assume a fixed task structure and to model-free RL based on Q-learning with soft-max action selection.

### Structure learning model with Bayesian reinforcement learning

In general, structure learning involves estimating the underlying dependency structure between variables. Such learning has been formulated as a probabilistic inference problem, where inference is performed over a family of hypothesized dependencies. Within machine learning, it is common to represent these dependencies using graphical models, in which nodes are variables and conditional dependencies between variables can be represented as edges.

**Figure 2:**
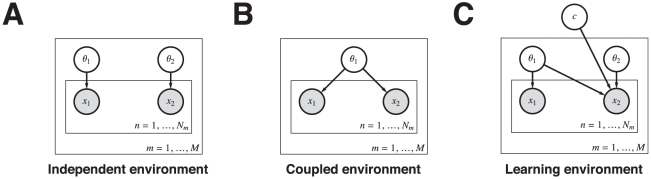
Graphical models of reward generation. The agent faces 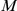 tasks, each comprising a random number 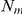 of choices. **A**) Rewarding options are independent. **B**) Rewarding options are coupled within a task. **C**) Mixture of tasks. Rewarding options may be independent or coupled. The node 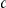 acts as a “XOR” switch between coupled and independent structure.

More specifically, a graphical model conveys knowledge on how a joint probability distribution can be factored into multiple known conditional probabilities. For example, in Fig. 2A, and ignoring all the plates, the edge from node 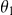 to node 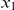 would indicate that the joint probability distribution 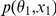 can be equivalently written as the product of two known distributions 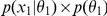. Additionally, a plate is a shorthand notation for replicating variables inside it while sharing conditional relationships and distribution functions. For example, the node 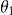 inside the plate with 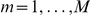 means that there are 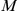 variables (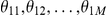) that have the same known distribution function. The node 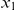 is inside a plate with 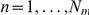 and inside the 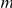 plate, which indicates—quite compactly—that the total set of nodes is 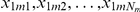 for each 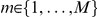. Finally, the conditional probabilities 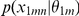, for any 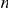 and 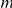, correspond to the same distribution function.

A variety of Machine Learning methods have been developed to perform structure learning in graphical models (e.g., [14], [15]), and these have provided a compelling account of human causal inference and learning in cognitive tasks [13], [16]. Below we show human structure learning in a sequential decision-making task. However, formulating the structure learning problem within sequential decision making is significantly more difficult, requiring a combination of probabilistic inference with reinforcement learning commonly called Bayesian reinforcement learning.

Bayesian reinforcement learning (BRL) can be used to describe an agent that learns the structure of rewards in the environment while performing optimal action selection that balances exploration and exploitation. Agents interact with a stochastic environment by performing an action 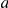 that affect the state of the environment 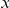 by transitioning to a new state 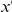 with probability 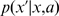. Rewards are received with a probability 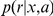 that depends on the action and the outcome of the action. For the agents we are interested in describing, the goal is to maximize the reward accumulated across participation in a set of tasks which end stochastically with a probability 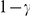. The optimal BRL agent schedules actions that maximize the expected reward received during the task: 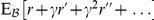, where 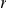 is the reward to be received immediately, 
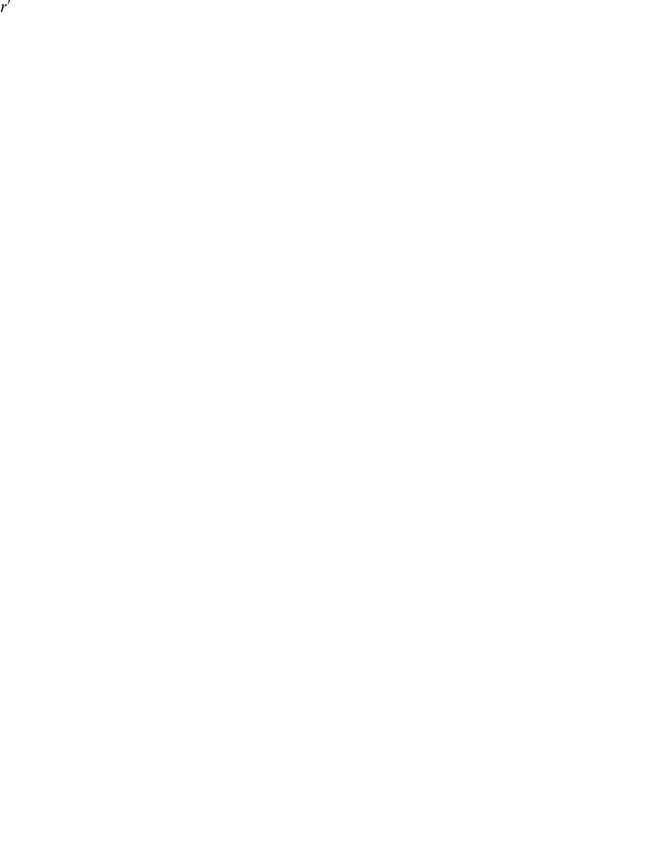
 the reward received next, 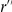 the reward received two steps into the future, and so on, and 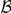 is current model of the environment. In standard model-based reinforcement learning, the agent uses a probabilistic model of reward sources and environment to compute this expectation. In BRL, the agent does not know either the reward sources and environment precisely, but rather generates *beliefs* over a family of possible models.

After each observation, the belief distribution is updated using Bayesian inference. By considering the set of possible future observations, this belief updating can be used to “look ahead” to predict future rewards that can be achieved from different plans of action. The *value of a belief* can be found using the Bellman equation [17]

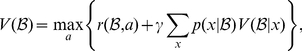
(1)where 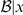 represents the belief “update” by Bayes' rule
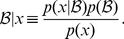
(2)


In the context of reinforcement learning, a policy is a prescription of what action should be taken at a particular state. One of the key ideas in BRL is that the optimal policy can be described as a mapping from belief states to actions. In particular, an optimal policy 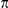 can be recovered by
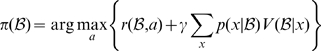
(3)


We specialized this framework to model structure learning in sequential decision experiments (see Materials and Methods for more details). For the BRL agent with structure learning, uncertainty about reward dynamics and contingencies can be modeled by including within the belief state not only reward probabilities, but also the possibility of independent or coupled structure. Maximizing the expected reward over this belief state yields the optimal balance of exploration and exploitation, resulting in action selection that seeks to simultaneously maximize (1) immediate expected rewards, (2) information about reward dynamics and (3) information about task structure.

Fig. 2A represents a graphical model for the generation of rewards in an independent environment. Rewards 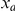 are samples from Bernoulli distributions with separate Beta prior distributed reward probabilities 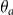 for each option. The belief state about 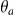 is summarized by the counts of the number of successes 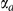 and failures 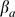 for each option. Fig. 2B shows a graphical model for a coupled environment. Coupling is represented as a “shared” probability of reward 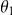 from which the rewards 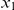 and 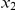 are sampled. However, the probability of reward 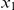 follows a Bernoulli distribution with parameter 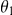 whereas 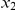 follows a Bernoulli distribution with parameter 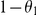.

To model learning coupling structure, we introduce a hidden binary state 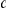, representing whether the options are independent or coupled in the environment. Uncertainty about the coupling structure generates a mixture between the independent and coupled environment models. Fig. 2C shows the full graphical model that incorporates uncertainty about the environment structure. It is a mixture model of the independent and coupled environments (Fig. 2A and B.) The parameter 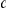 switches between a coupled environment for 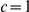 and an independent environment for 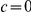 (see Materials and Methods for details.). Structure uncertainty is captured by a Bernoulli distribution on 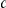 with parameter 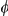, which will change solely based on the rewards observed.

Without uncertainty, the optimal decision-making strategies for both the independent and coupled environments are well-known and relatively simple. The optimal policy for a coupled environment is purely exploitative—it simply chooses the option with the greater number of successes (including failures of the other option as successes) because the reward observed in one option tells us *everything* the reward that would have been received in the other option. Optimal action selection for an independent environment, however, involves balancing the exploration–exploitation trade-off. Exploration is required because choosing one option does not provide information about the other. The optimal policy for an independent environment involves computing a quality index for each option, called the Gittins index [18], and selecting the highest quality option. The Gittins index computes the maximum expected reward per unit discounted time for each option, and is the result of the following optimization problem:
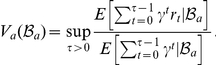
With uncertainty, optimal action selection depends on the belief that the environment is coupled, as captured by the parameter 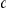. In the methods section, we show that the optimal policy for structure learning can be expressed as a mixture of the optimal policies for the independent and coupled environments. For all the models, the optimal policy 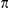 is a function of the observed counts of successes, 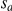, and failures, 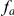, for each option, and priors 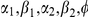.

**Figure 3:**
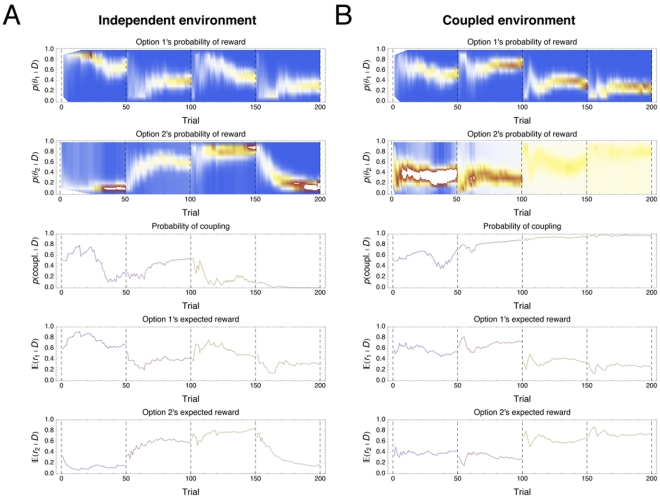
Learning simulation of structure learning model. Four tasks of 50 trials each are sequentially shown to the structure learning model. Priors were 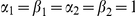 and 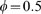. Marginal beliefs on reward probabilities (brightness indicates relative probability mass), probability of coupling and expected reward are shown as functions of time. **A**) Simulation on Independent Environment **B**) Simulation on Coupled Environment.

To illustrate the behavior of the structure learning model, we expose the model to a sequence of tasks. The model is placed in either a coupled or independent environment (Fig. 3A & B). Every 50 trials the reward probabilities on the options are randomly reset, but the type of environment stays fixed. For both environments, the structure learning model learns the environment type, as expressed by the convergence of the posterior distribution on the 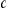 parameter to its true value. For the parameters 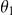 and 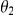, the marginal probability is indicated by the color, with brighter indicating higher relative probability mass. The structure learning model quickly learns in both environments, although it is frequently easier to detect an independent environment—whenever both options are significantly above or below chance, the coupled structure can be quickly ruled out. Once there is high certainty on the structure (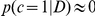 or 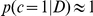, where 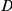 is the data), beliefs are concentrated on the parameters that matter for that structure—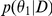 and 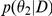 becomes concentrated on the reward probabilities of each option in the independent environment, and 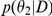 becomes uniform in the coupled environment.

**Figure 4:**
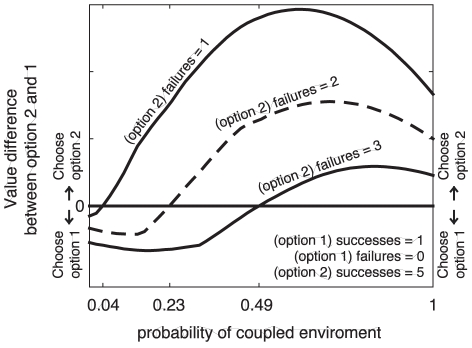
Effect of task uncertainty on exploration– exploitation of structure learning model. The data available for the options are 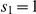, 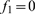, and 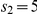 and discount factor 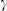 is 0.98, all values fixed for the simulation. The number of failures for option two (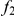) is varied from 1 through 3. Under these conditions, the independent would always choose option 1 whereas the coupled model would always choose option 2. However, the structure learning model switches between these two The graph shows the difference in values between the option 2 and 1 as a function of the task uncertainty.

The effect of structure uncertainty on the behavior of the structure learning model is evident by looking at the expected reward. For action 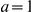, this expected reward is
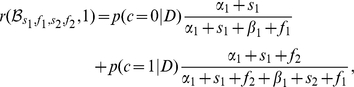
where 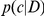 is the posterior probability on the structure given the data 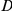 represented by the counts 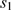, 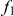, 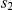 and 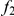. If the probability that the structure is coupled is high (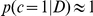), then the expected reward accrues regardless of which action is chosen. If the probability that the structure is independent (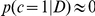) is high, then the expected reward depends only on the option chosen. Thus the belief about coupling gates the need for exploration. In an independent model, there is a value attached to choosing the option with less evidence even if the current evidence suggests it has a lower probability of success. The expected reward for action 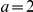 is similarly
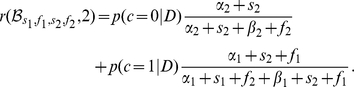
In Fig. 4, we perform a simulation that shows how the structure learning model described can behave as a independent or coupled model depending on the uncertainty about coupling belief. We purposely chose evidence values for which the independent model would pick one option while the coupled model would pick the other. When a curve dips below 0, it means that the learning model would choose option 1, and when it does above 0, it would pick option 2. Note that the structure learning model can sometimes behave as a coupled or independent model depending on the uncertainty about the structure. This difference between the structure learning model vs. fixed models will play an important role later when we show that people change their policy in accord with structure learning.

### Model comparison

To quantify structure learning in participant's decisions, we compared the predictions of the structure learning model with models that capture the decisions expected from knowledge of structure in the absence of learning (fixed independent and coupled structure). Additionally, we used Q-learning algorithm [19] with a soft-max action selection [20] as a base model. Q-learning is a model-free RL method that does not model the reward probabilities or structure, rather it estimates the value of an action by compiling over experienced outcomes (called Temporal Difference learning). However, Q-learning does not balance exploration and exploitation in a principled way, but rather performs heuristic explorations based on random actions. It is proven to estimate the optimal value of an action after infinitely many observations for every action and state [19]. The temporal difference aspect of Q-learning as well as the exploratory interpretation of the soft-max rule have been shown to correlate with brain activity [4], [21], [22].

Fitting the models to all the response data, we find that the structure learning model prediction rate (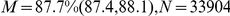) is better than the coupled model prediction rate (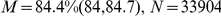), exact binomial test 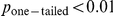, better than the fixed independent model prediction rate (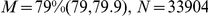), 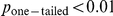, and better than Q-learning model (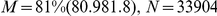), 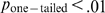). Note that the Bayesian models have no free parameters, with the exception of the initial value of the prior belief about coupling structure 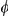 for the structure learning model, which is quickly swamped by the evidence. However, we allowed for individual differences in all five parameters of the Q-learning model. For all models, we assumed uniform priors on probabilities of reward (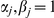, 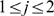, at the beginning of tasks).

The remainder of the results are organized as follows. Because essentially all models predict well a large number of trials that occur later in blocks (where evidence is high and the better option is easy to identify), we focus on the set of trials for which there is at least one disagreement between the models so that we can better tell them apart. We call this set of trials *diagnostic*. We show the structure learning model can better account for several aspects of decision-making on diagnostic trials. In particular, we show how uncertainty in task structure tracks qualitative and quantitative changes in choice behavior. Then we show that the structure learning model gives a principled explanation for strategies that appear suboptimal. Finally, we analyze decisions that are specifically diagnostic for the structure learning model (structure learning predicts differently than fixed models) and show that the structure learning model predicts human choice behavior better than models with fixed structure.

#### Participants' decisions better captured by a structure learning model

We show 1) participants quickly adapt their choices to the environment that they are in, independent or coupled, and 2) normative belief about coupling predicts participants exploratory moves while learning which type of environment they are in.

Because optimal policies depend on the observed rewards for both options, we analyzed participants' choices as a function of two measures of the observed successes and failures: evidence and confidence. In essence, we categorized a trial based on the observation history that preceded it. The evidence measure is the log odds ratio of the observed reward rate of the better option (higher reward probability) to the worse (lower reward probability).
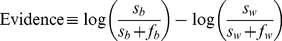
(4)where 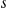 and 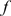 denotes the observed number of successes and failures respectively and the subscripts 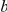 and 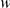 denote the better and worse options, respectively. The confidence measure is the log of the ratio of the number of observations at each option
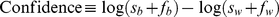
(5)Together the two measures capture the important aspects of the observed successes and failures for decision-making, and are commonly used to analyze proportional data [23]. Evidence measures which option appears better (in relative terms) based on the observed frequencies. Confidence measures the relative reliability of the evidence.

**Figure 5:**
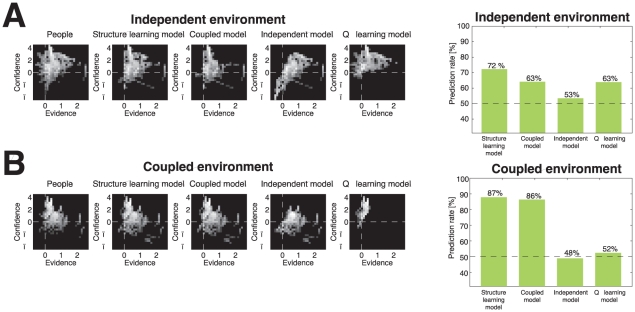
Full behavior on diagnostic trials as a function of evidence and confidence. Diagnostic trials are those in which there is at least one disagreement between the models. For each of these trials, we compute the evidence and confidence of each option. A cell in the graph indicates the empirical probability that the model (or participants) pick the better option as a function of evidence and confidence. The right panels show prediction rate of different models in diagnostic trials. All pair-wise differences are significant (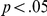) **A**) Trials in Independent Environment **B**) Trials in Coupled Environment.

We compile all choices in the diagnostic trials with the same evidence and confidence and computed the fraction of these choices to the better option. We separated our analysis for the independent environment (Fig. 5A, left panel) and coupled environment (Fig. 5B, left panel). Multiple pair-wise comparisons between the models reveal that the structure learning model is significantly better at predicting participants' decisions than the rest of the models, 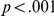 (Fig. 5A and B, right panels)

#### Participants' choices are tracked by structure uncertainty of structure learning model

**Figure 6:**
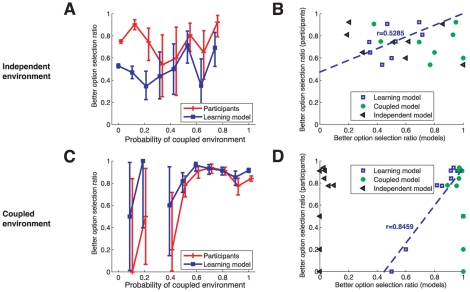
Better arm selection ratio. In the diagnostic trials, **A**) and **C**) Belief in coupling tracks changes in participant choices similarly to the learning model **B**) and **D**) behavior vs. structure belief is well correlated with the learning model, but not with independent and coupled.

To better test whether participants' decisions reflect structure learning, we analyze how coupling belief affected decisions within diagnostic trials. For each trial, we computed the learning model's coupling belief for the sequence of observed rewards (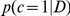, where 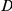 is the reward history). We then computed the fraction of choices to the better option as a function of coupling belief, for both participants and for each of the models. The results are shown in Fig. 6A,B. Qualitatively, human choices mirror the structure learning model. Quantitatively, the structure learning model correlates strongly with participants in the coupled environment (Fig. 6D), 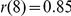, 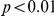, and less on the independent environment (Fig. 6B), 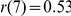, 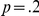. However, the correlation to fixed models is weaker in both environments (independent environment: 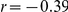 independent model, 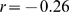 coupled model; coupled environment: 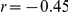 independent model, 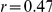 coupled model.). Taken together, these results suggest that people are behaving remarkably like an optimal structure learning model in a couple environment, with some unaccounted behavior in an independent environment.

#### Behavior deemed suboptimal by fixed structure models are optimal for structure learning

In the following sections, we focus on explaining trials that are deemed suboptimal if the process of reward generation of the environment is assumed known by the participant. In particular, we show that uncertainty about task structure provides incentive for making these apparently sub-optimal choices.

Some studies have suggested that behavior in two independent option tasks is suboptimal when compared to an optimal model [24]–[27] —that people explore too little to find the better option quicker, or explore too much, continuing to choose an option that should have been discarded. We tested whether these types of trials are better predicted by the learning model.

**Figure 7:**
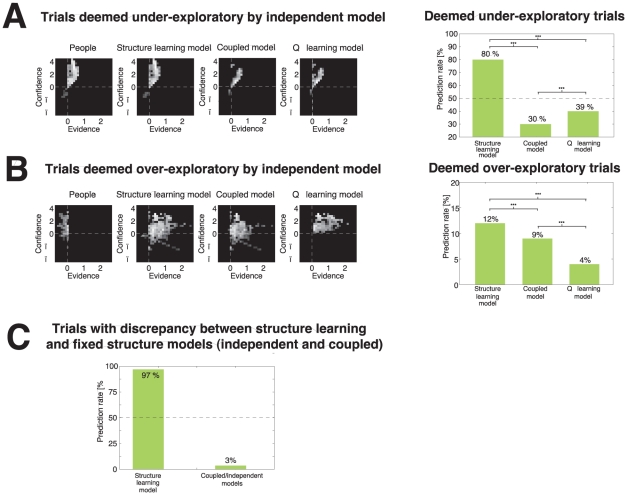
Model comparison in different aspects of decision-making. **A** and **B**) Performance of learning model and coupled model for decisions not predicted by the independent model in the independent environment (separated into *under-exploratory* and *over-exploratory* trials) **C**) Prediction performance for trials where independent and coupled model prefer one option whereas the learning model prefers the other. These trials are called *task learning* trials.

By under-exploration, we mean that subjects choose differently than an independent model for trials where the independent model selects the option with lower reward proportion (because the counts are low), and thus the independent model has a higher value for the lower reward probability option. By over-exploration, we mean that subjects choose differently than an independent model for trials where the independent model selects the option with higher reward proportion and high counts—i.e., the option chosen is clearly less rewarding and there should be nothing left to learn from it. A percentage of trials are under-explorative (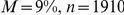) or over-explorative (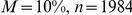) out of the number of trials in the independent environment (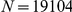). The learning model was able to predict most of the under-exploratory trials (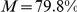 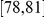), and significantly more trials than other models, 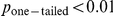 (see Fig. 7A). The learning model also predicted over-exploratory trials (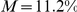 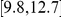) better than the other models, 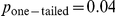, but the predictive performance is relatively poor (Fig. 7A).

The subset of trials classified as over-exploratory by the independent model were not well predicted by any of the models, which essentially corresponds to an anti-diagonal trend in participants decisions in the evidence versus confidence space (see Fig. 6, left panel). For negative evidence and positive confidence and for positive evidence but negative confidence, participants choose opposite to normative predictions. Both of these cases correspond to participants persisting in choices despite evidence to the contrary. We believe that this pattern may be a consequence of temporal dependence in participants choices, a possibility we return to in the Discussion section.

Behavior in coupled environments has also been suggested to be sub-optimal [24], [25], [28]–[31]. Given that there is no need for exploration and the optimal behavior is inherently exploitative, we tested whether behavior that diverged from the coupled model's predictions would be better predicted by the learning model. A small percentage of trials (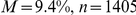) disagreed with the coupled model in the coupled environment (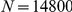). The learning model predicts 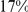 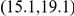 of these trials, and has higher prediction rate than the independent model, although not significant, 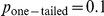.

#### Trials not predicted by the coupled or independent models are task-learning trials predicted by structure learning model

For structure learning tasks, there are decisions purely intended to diminish the uncertainty about the structure. A simple way to isolate these decisions is by selecting trials in which fixed models (coupled and independent) pick one option while the structure learning model picks the other. A Welch-Satterthwaite two-sample 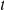-test confirms the intuition that these trials happen earlier than other trials within an environment, 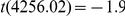, 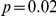. For these trials, the learning model was able to predict almost all of participants' decisions (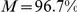 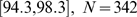), and thus the fixed models predicted almost none (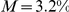 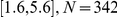), exact binomial test 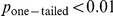 (see Fig. 7C). Q-learning predictions were also worse than chance on these trials 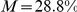 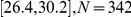), and worse than structure learning model 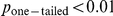.

## Discussion

We have provided evidence that structure learning may be an important missing piece in evaluating human sequential decision making. The idea of modeling sequential decision making under uncertainty as a structure learning problem is a natural extension of previous work on structure learning in models of cognition [13], [16] (also see [32]), animal learning [33] and motor control (e.g., see [34]). It also extends previous work on Bayesian approaches to modeling sequential decision making in the Multi-armed bandits [35] by adding structure learning. It is important to note that we have intentionally focused on reward structure, ignoring issues involving dependencies across trials. Clearly reward structure learning must be integrated with learning about temporal dependencies [36] (e.g. assumptions of a non-stationary environment [5], [37], [38]).

Interestingly, there were a set of participants' decisions that none of the models were able to capture and that constitute 9.4% of the data. These trials are predominately localized on positive evidence (Eq. 4), but negative confidence (Eq. 5) levels (see Fig. 5A and B, left panel, people column.). These choices corresponded to persisting in choosing the worst option despite statistical evidence supporting the better option. None of the models considered would choose the worse option under these conditions. Participants may have limited memory or may be considering a larger space of possible models; for example nonconstant reward rates (allowing for nonstationary reward probabilities).

Although we focused on learning coupling between options, there are other kinds of reward structure learning that may account for a broad variety of human decision making performance. In particular, allowing dependence between the probability of reward at a site and previous actions can produce large changes in decision making behavior. For example, in a “foraging” model where reward is collected from a site and probabilistically replenished, optimal strategies will produce choice sequences that alternate between reward sites [39]. Thus uncertainty about the independence of reward on previous actions can produce a continuum of behavior, from maximization to probability matching. Note that structure learning explanations for probability matching are significantly different than explanations based on reinforcing previously successful actions (the “law of effect”) [40]. Instead of explaining behavior in terms of the idiosyncrasies of a learning rule, structure learning constitutes a fully rational response to uncertainty about the causal structure of rewards in the environment. By expanding the range of normative hypotheses for human decision-making, we believe we can begin to develop more principled accounts of human sequential decision-making.

The general alternative to the rational approach is to assume that choice behavior reflects some fundamental limitations in sensing, neural computation or storage. It is possible that the decisions we could not predict in any dependent environment result from human processing limitations. For example, one of the key decision patterns that does not fit in the normative approach is choice stickiness, a persistence in choosing the same option despite evidence suggesting it would be better to switch. This could reflect a transition to model-free learning in the independent environment. Participants may have learned a policy for choosing that option based on early reward evidence. However, we find no evidence for this possibility in our data. Another possibility is that participants have memory limitations that prevent them from compiling all of the evidence [35]—the observed persistence may be sensitivity to local reward. While limitations to human decision-making surely exist, and people are bounded rational, our results provide evidence that decisions are also driven by sophisticated structure learning. We believe that many aspects of human decision-making that appears mysterious may be the result of the brain's attempts to acquire compact and useful representations of the structure of its environment.

We foresee an adoption of more sophisticated models of sequential decision-making to account for the compact representation that humans might be using to act in diverse reward structures. While we believe that the theory to analyze these representations is available, it has only been cautiously adopted in Psychology and Neuroscience [35], [41]–[43]. We have already seen this pattern of adoption occur in Artificial Intelligence where the development of efficient computational methods to solve Bellman's equation (i.e. model-free RL methods like Q-learning) led to the rapid development and application of RL methods starting in the 1980s, despite the fact that the theoretical foundations had been laid by control theorist more than two decades prior [1], [44], [45]. While Robotics, for example, today hardly uses model-free reinforcement learning to think about tasks of any level of complexity, much work remains for model-based reinforcement learning to make its way into mainstream human and animal sequential decision-making analysis.

## Materials and Methods

Informed consent was obtained and all investigations were conducted according to the principles expressed in the Declaration of Helsinki, under the Assurance of Compliance number FWA00000312.

### Experimental methods

Sixteen volunteers solve 32 bandit tasks, 16 for each environment. The probabilities of rewards were randomly sampled from a uniform distribution, and the stopping times for each bandit task were sampled from a Geometric distribution 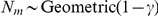. The average stopping time was 48. The order of the tasks within an environment was randomized, and the order of presentation of the environments was randomized as well. All subjects were exposed to the same probabilities of rewards and stopping times.

Each option is shown in the screen as a slot machine. Subjects pull a machine by pressing a key in the keyboard. When pulled, an animation of the lever is shown, 200 msec later the reward appears in the machine's screen, and a sound mimicking dropping coins lasts proportionally to the amount gathered. We provide several cues, some redundant, to help subjects keep track of previous rewards. We display the number of pulls, total reward, and the current average reward per pull. Reward magnitudes were 0 or 100 points. The machine's screen changes the color according to the average reward, from red (zero points), through yellow (fifty points), and green (one hundred points). The machine's total reward is shown as a pile of coins underneath it. The total score, total pulls, and rankings within a game were presented.

All participants finished all tasks. Each participant performed 1194 trials on independent environment and 925 on the coupled environment, for a total of 33904 trials. In general, participants understood the task well. No apparent outliers were found nor missed trials.

### Models of sequential decision-making

The language of graphical models provides a useful framework for describing the possible structure of rewards in the environment. Consider an environment with several distinct reward sites that can be sampled, but the way models generate these rewards is unknown. In particular, rewards at each site may be independent, or there may be a latent cause which accounts for the presence of rewards at both sites. Uncertainty about which reward model is correct naturally produces a mixture as the appropriate learning model. This structure learning model is a special case of Bayesian Reinforcement Learning (BRL), where the states of the environment are the reward sites and the transitions between states are determined by the action of sampling a reward site. Uncertainty about reward dynamics and contingencies can be modeled by including within the belief state not only reward probabilities, but also the possibility of independent or coupled rewards. Then, the optimal balance of exploration and exploitation in BRL results in action selection that seeks to maximize (1) expected rewards (2) information about rewards dynamics, and (3) information about task structure.

The belief over dynamics is effectively a probability distribution over possible Markov Decision Processes that would explain observables. As such, the optimal policy can be described as a mapping from belief states to actions. In principle, the optimal solution can be found by solving Bellman optimality equations but generally there are countably or uncountably infinitely many states and solutions need approximations. If we were certain which of the two models were right, the action selection problem has known solution for both cases, presented below.

#### Model with fixed independent structure

Learning and acting in an environment like the one described in Fig. 2A is known as the Multi-Armed Bandit (MAB) problem. The MAB problem is a special case of BRL because we can partition the belief 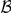 into a disjoint set of beliefs about each option 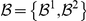. Because beliefs about non-sampled options remain *frozen* until sampled again, independent learning and action selection for each option is possible. Let 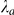 be the reward of a deterministic option in
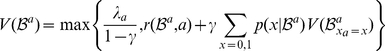
such that both terms inside the maximization are equal. Gittins [18] proved that it is optimal to choose the option 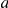 with the highest such reward 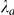 (called the Gittins Index). This allows speedup of computation by transforming a *many*-arm bandit problem to *many* 2-arm bandit problems.

In our task, the belief about a binary reward may be represented by a Beta Distribution with sufficient statistics parameters 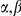 (both 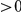) such that 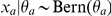, where 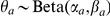. Thus, the belief about option 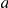 is 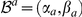 expected reward 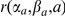 and predicted probability of reward 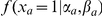 are 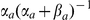. The belief state transition is 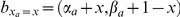. Therefore, the Gittins index may be found by solving the Bellman equations using dynamic programming
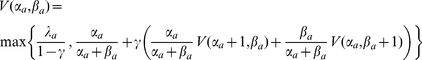
(6)to a sufficiently large horizon. In experiments, we use 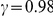, for which a horizon of 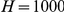 suffices.

#### Model with fixed coupled structure

Learning and acting in coupled environments (Fig. 2B) is trivial because there is no need to maximize information in acting. The belief state is represented by a Beta distribution with sufficient statistics 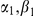 (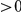). The expected reward of option 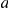 is then defined as:
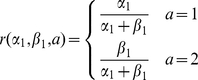
(7)


The optimal value of action is myopic as follows
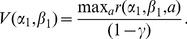
(8)


The belief state transitions are 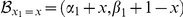 and 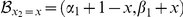.

#### Learning and acting with structure learning model

We restrict ourselves to the following scenario. The agent is presented with a sequence of 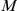 bandit tasks, from 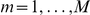, each with initially unknown Bernoulli reward probabilities and coupling. Each task involves 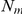 discrete choices, where 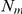 is sampled from a Geometric distribution with parameter 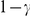.

Fig. 2C shows the mixture of two possible reward models shown in Fig. 2A and B. Node 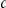 switches the mixture between the two possible reward models and encodes part of the belief state of the process. Notice that 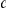 is acting as a *XOR* gate between the two generative models. Given that it is unknown, the probability distribution 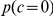 is the mixed proportion for independent reward structure and 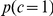 is the mixed proportion for coupled reward structure. Specifically:

1. For the block: Coupling parameter 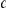 may be either 0 or 1, and is unknown for the agent. For learning, put Bernoulli prior with parameter 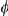. Sample 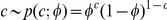.
2. For the bandit task 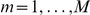: Sample 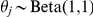 for parameters, all unknown for the agent. For learning, put Beta priors 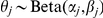, with 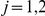.
3. For choice 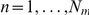, with stochastic stopping time 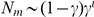:

- Choose option 1: 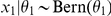

- Choose option 2: 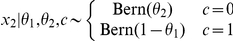

Learning can be performed analytically. Let **x** be a sequence of rewards observed. For the likelihood term 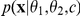 in the posterior, the observations **x** are independent given 
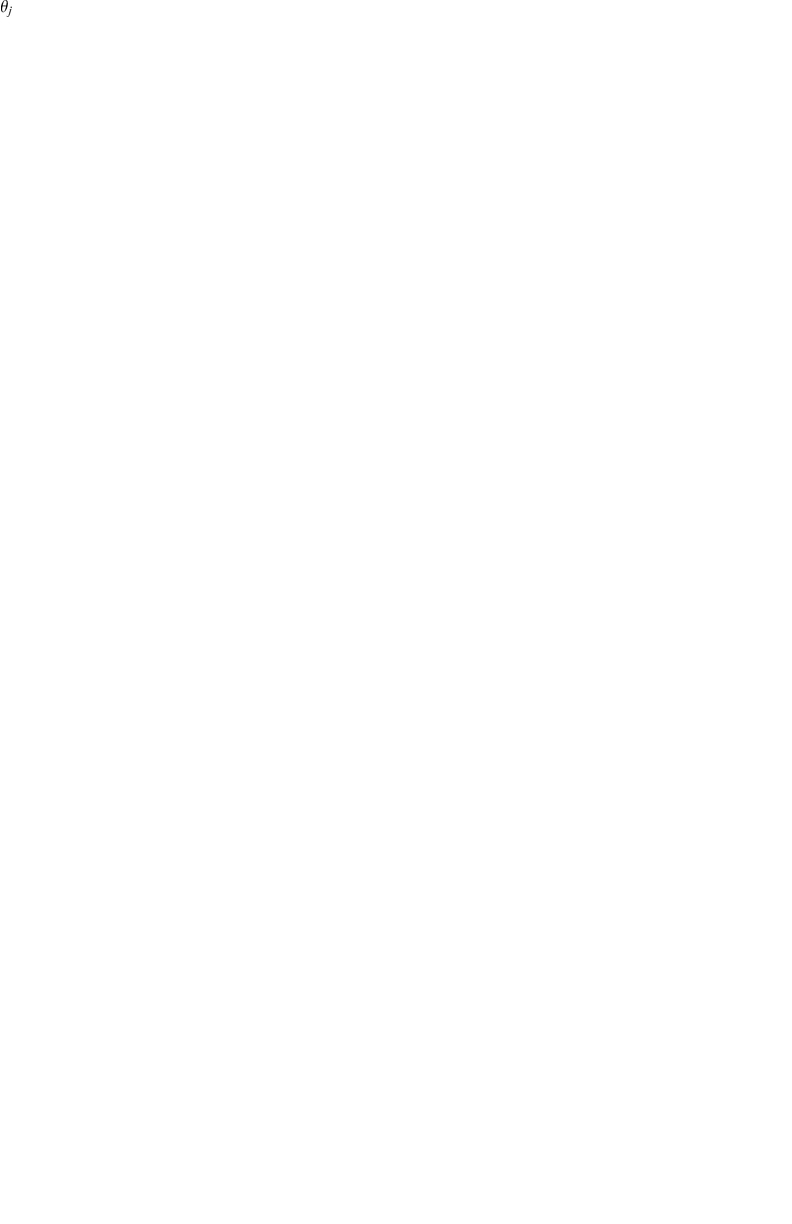
's and 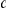. Hence, we just need to keep track of the number of successes (1's) and failures (0's) of each option, rather than *when* rewards were observed. Let 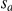 and 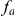 be the number of successes and failures for option 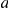 in **x**. It is clear that the posterior distribution 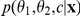 is not closed with respect to the prior, but still by keeping track of the counts we can compute the necessary quantities for the Bellman's equation in a straightforward manner.

After simple algebraic manipulation, we can obtain the posterior distribution on coupling. At the beginning of each bandit task, we assume the agent “resets” its belief about options (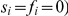, but the posterior over 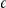 is carried over and used as the prior on the next bandit task. Let 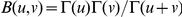 be the Beta function, where 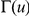 is the Gamma function. For simplicity, we define 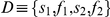. The marginal posterior on 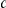 is as follows
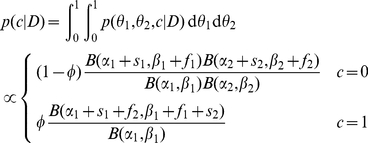
(9)


The beliefs about environment dynamics, however, may still be completely represented by the counts and prior parameters within a task with a probability distribution about environment dynamics as Eq. 9.

The predicted rewards are:
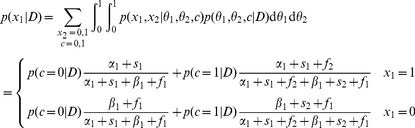
(10)and similarly
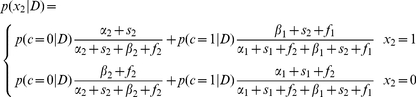
(11)


From now on, we define 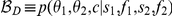 for simplicity. The action selection involves solving the following Bellman equations
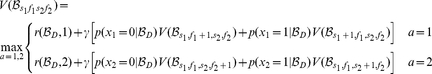
(12)


To obtain (12) using dynamic programming for a horizon 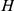, there will be a total of 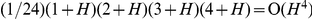 computations which represent different occurrences of 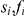 out of 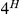 possible histories of rewards. This dramatic reduction allows us to be relatively accurate in our approximation to the optimal value of an action.

We use a horizon 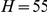 for computing values with Eq. 12. Notice that we can recover the action selection of fixed models by computing 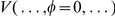 for the independent model and 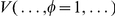 for the coupled model. However, we use Eq. 6 for the independent model and Eq. 7 for the coupled environment because is much more efficient. We checked that actions of the learning model when the task certainty is very high (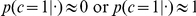) do not differ from Eq. 6 or Eq. 7, respectively.

#### Q-learning with soft-max

It is possible to optimally act without a model of the environment by using what is known as model-free reinforcement learning. One of the most popular model-free reinforcement learning algorithms is known as Q-learning, which can compute the optimal value of an action after infinitely many observations for each action and states [19]. However, Q-learning does not have a principle for performing exploratory actions and it is usually coupled with occasional random actions (e.g., see [10], [12] for a contrast with Bayesian reinforcement learning). For example, the 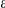-greedy action selection chooses a random action an 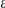 fraction of the time and the soft-max action selection uses the current estimates of values to construct a distribution on the probability where, roughly speaking, actions with higher value estimates have higher probability of selection. In practice, 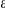-greedy and soft-max Q-learning are extremely fast methods for making decisions, but they do not keep track of the accuracy and need a great deal of data to correctly estimate values.

We use Q-learning with soft-max action selection as model for base comparison. Suppose that the value of each option at time 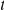 is 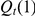 and 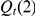, then the action selection is random and driven by the following soft-max rule:
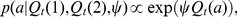
(13)where 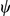 has the following interpretation: a large value (e.g., 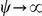) indicates that the agent will always choose the option with highest 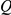, a value 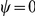 indicates that the agent will pick an option uniformly at random, and a negative value (e.g., 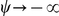) indicates that agent tends to choose in opposition to what is prescribed by the Q values.

After taking an action 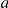, interacting with the environment and receiving a reward 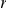, the agent updates its estimation of the values by the temporal difference rule:
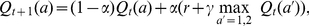
(14)where 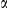 is known as the *learning rate* and 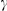 is the discount factor. A learning rate 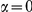 indices that the agent won't consider new rewards in the estimation of 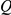, while a learning rate 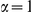 indicates that the agent will consider only the last reward in the estimation and not past rewards.

Q-learning needs an initial estimation of the value of each option (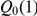 and 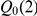), the learning rate 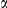 and the parameter 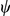 for the soft-max rule. For our data analysis, we fit these parameters per participant so as to maximize the prediction rate of the Q-learning model.
